# Prevalence of *Yersinia* Species in the Ileum of Crohn's Disease Patients and Controls

**DOI:** 10.3389/fcimb.2018.00336

**Published:** 2018-09-21

**Authors:** Guillaume Le Baut, Claire O'Brien, Paul Pavli, Maryline Roy, Philippe Seksik, Xavier Tréton, Stéphane Nancey, Nicolas Barnich, Madeleine Bezault, Claire Auzolle, Dominique Cazals-Hatem, Jérome Viala, Matthieu Allez, The REMIND GROUP, Jean-Pierre Hugot, Anne Dumay

**Affiliations:** ^1^UMR1149 INSERM, Research Centre on Inflammation, Université Paris Diderot-Sorbonne Paris-Cité, Paris, France; ^2^Department of Gastroenterology and Nutrition, Centre Hospitalier Universitaire de Caen, Caen, France; ^3^IBD Research Group, Canberra Hospital, Canberra, ACT, Australia; ^4^Australian National University Medical School, Canberra, ACT, Australia; ^5^Gastroenterology Unit, CNRS, INSERM, ERL 1157, LBM, APHP, Saint Antoine Hospital, Sorbonne Universités, UPMC Univ Paris 06, Ecole Normale Superieure, Paris, France; ^6^Departments of Gastroenterology and Pathology, Hôpital Beaujon, Assistance Publique Hopitaux de Paris, Paris, France; ^7^Department of Gastroenterology, Hospices Civils de Lyon, Lyon-Sud Hospital, Pierre-Bénite, France; ^8^INSERM U1111, International Center for Research in Infectiology, Lyon, France; ^9^UMR 1071 Inserm/Université d'Auvergne, USC-INRA 2018, Microbes, Intestin, Inflammation et Susceptibilité de l'Hôte (M2iSH), CRNH Auvergne, Clermont-Ferrand, France; ^10^Department of Gastroenterology, Saint-Louis Hospital, APHP, INSERM U1160, University Denis Diderot, Paris, France; ^11^Department of Pediatric Gastroenterology, Hôpital Robert Debré, Assistance-Publique Hôpitaux de Paris, Paris, France

**Keywords:** *yersinia*, Crohn's disease, gut microbiota, ileal mucosa, innate immunity, mucosal immune system, molecular test

## Abstract

*Yersinia* are common contaminants of food products, but their prevalence in the human gut is poorly documented. *Yersinia* have been implicated in Crohn's Disease (CD, an inflammatory bowel disease) however their role in CD is controversial. We performed highly sensitive PCR assays of specific sequences for the *gyrB* gene of *Y. aldovae, Y. bercovieri, Y. enterocolitica, Y. intermedia, Y. mollaretii* and the *inv* gene of *Y. pseudotuberculosis*. We analyzed a total of 470 ileal samples taken from 338 participants (262 CD patients and 76 controls) belonging to three independent cohorts. All patients and controls were phenotyped and genotyped for the main CD susceptibility variants: *NOD2, ATG16L1*, and *IRGM*. *Yersinia* were found in 7.7% of ileal samples (respectively 7.9 and 7.6% in controls and CD patients) corresponding to 10% of participants (respectively 11.8 and 9.5% in controls and CD patients). *Y. enterocolitica, Y. pseudotuberculosis* and *Y. intermedia* were the most frequently identified species. The bacteria were more frequent in resected specimens, lymph nodes and Peyer's patches. *Yersinia* were no more likely to be detected in CD tissues than tissues from inflammatory and non-inflammatory controls. CD patients treated with immunosuppressants were less likely to be *Yersinia* carriers. In conclusion, this work shows that *Yersinia* species are frequently found at low levels in the human ileum in health and disease. The role of *Yersinia* species in this ecosystem should now be explored.

## Introduction

The genus *Yersinia* comprises 18 species, 3 of which are well-recognized human pathogens: *Y. pestis*, the causative agent of plague, and the two enteropathogenic species *Y. pseudotuberculosis* and *Y. enterocolitica*. The 15 other species are *Y. aldovae, Y. aleksiciae, Y. bercovieri, Y. entomophaga, Y. frederiksenii, Y. intermedia, Y. kristensenii, Y. massiliensis, Y. mollaretii, Y. nurmii, Y. pekkanenii, Y. rohdei, Y. ruckeri, Y. similis*, and *Y. wautersii*.

Various culture-dependent, immunological, and molecular techniques are currently available for the detection of pathogenic *Yersinia* (Gupta et al., 2015). The presence of *Y. enterocolitica* and *Y. pseudotuberculosis* can be determined quantitatively by direct culture on selective agar plates but confirmatory tests require a combination of cold enrichment and subcultures. Culture-independent methods have been developed to detect pathogenic *Y. enterocolitica* and *Y. pseudotuberculosis*. These PCR assays usually target the *yadA* or *virF* gene located on the pYV plasmid, and thus only detect virulent strains. Consequently, little is known about the presence of non-pathogenic *Yersinia* species in most of studied samples. *Yersinia* prevalence in food products, especially for low virulence species is thus undoubtedly underestimated, especially considering that *Yersinia* surveillance is not systematic in industrial processes.

Despite these limitations, *Yersinia* (mainly *enterocolitica*) have been associated with a variety of foods, including milk and milk products, raw meat (beef, pork, chicken, and lamb), poultry, eggs, vegetables, bean sprouts, tofu, seafood, and others. *Yersinia* species are able to propagate in vacuum-packed foods and at refrigeration temperature. Refrigeration of contaminated foods at manufacturing and consumer sites may provide *Yersinia* species an opportunity to survive and thrive in food. A recent shift toward the increased consumption of processed foods wherein contamination can occur after pasteurization has also potentiated the risk of outbreaks. Expansion in international food trade and changes in livestock farming and food industry have also led to the emergence of yersiniosis globally (Gupta et al., 2015).

As an example, a recent study performed in Turkey found 84 (28%) out of 300 food samples were contaminated by *Yersinia* species (Özdemir and Arslan, 2015). Of the food samples analyzed, 41% of 120 meat products and 19% of 180 milk products contained *Yersinia* species. *Y. enterocolitica* was the most frequently detected species (*N* = 18), followed by *Y. rohdei* (*N* = 15), *Y. intermedia* (*N* = 14), *Y. pseudotuberculosis* (*N* = 12), *Y. ruckeri* (*N* = 12), *Y. mollaretii* (*N* = 5), and *Y. bercovieri* (*N* = 4). In another study from Austria, 90/120 (75%) of meat or fish samples contained *Yersinia* (Hilbert et al., 2003). In France, *Yersinia* was detected in eggs, vegetables, pastries, and others (Le Guern et al., 2016). Exposure to *Yersinia* in our food products appears to be common across continents.

Following food ingestion, about 10% of bacteria survive the acidic gastric environment. If they do survive, they may translocate the gut barrier via the follicle-associated epithelium, which comprises Peyer's patches in the small bowel, and isolated lymphoid follicles in the large bowel. Following translocation, *Yersinia* may be phagocytosed by macrophages and drain to neighboring lymph nodes via lymphatic vessels, and less often to the portal blood stream.

It has been proposed that *Yersinia* species may contribute to the occurrence or persistence of gut inflammation in Crohn's Disease (CD) patients (Hugot et al., 2003), which is a chronic relapsing inflammatory condition of the digestive tract. CD can occur anywhere along the gastrointestinal tract, but most commonly affects the terminal ileum. Like yersiniosis, CD inflammation is initially focused around the follicle-associated epithelium (Fujimura et al., 1996; Krauss et al., 2012) and may progress into deeper, more diffuse ulcerations. Lymphatic vessels have been shown to play a key role in the dissemination in both yersiniosis (Von Der Weid and Rainey, 2010) and CD (Randolph et al., 2016). CD is characterized by an increased and persistent reactivity against the gut microbiota and similar observations have been made in mice infected with *Yersinia* (Fonseca et al., 2015). Several *NOD2* gene variants and a variant on the *ATG16L1* gene are strongly associated with CD. In mice, *Nod2* mutations homologous to the human CD-associated mutations favor the survival of animals orally infected with *Y. pseudotuberculosis* (Meinzer et al., 2012). Similarly, the CD-associated *Atg16l1* mutation has been associated with decreased clearance of *Y. enterocolitica* and a concurrent increase in inflammatory cytokine production (Murthy et al., 2014).

*Yersinia* infection in CD patients has been explored using conventional methods. Sera from CD patients were found to be reactive to enteropathogenic *Yersinia* more often than control sera (Saebo et al., 2005). Five studies used *Yersinia*-specific sequence primers to detect *Yersinia* species in CD tissues (Kallinowski et al., 1998; Lamps et al., 2003; Knösel et al., 2009; Chiodini et al., 2013; Leu et al., 2013). Overall 0–63% of CD samples were positive compared to 0–31% of inflammatory or non-inflammatory controls. In all studies, *Yersinia* were found in CD and/or controls suggesting that *Yersinia* is a common inhabitant of the human gastrointestinal tract. Differences in patient and control cohorts, tissues analyzed, and PCR methods may explain the large variations reported. Of note, Knosel et al. found more *Yersinia* species in control than CD archived tissues. In their study, PCR signals were weak suggesting that a non-specific sequence may have been amplified or alternatively that contaminations may have occurred. In none of the above studies, except for Chiodini's work, were the PCR products sequenced in order to confirm their specificity.

To further document the presence of *Yersinia* in intestinal tissues of CD patients and controls, we analyzed samples from three cohorts, representing a total 470 samples from 338 participants. All patients and controls were phenotyped and genotyped for the most strongly associated CD susceptibility gene variants. All samples were analyzed under the same conditions, using a validated sensitive PCR method capable of detecting six *Yersinia* species. PCR products were systematically sequenced to confirm the presence of *Yersinia*. Correlations between *Yersinia* positivity and clinical or genetic parameters were assessed.

## Materials and methods

### Patients

Three cohorts were used in this study. Cohort 1 is an Australian cohort of 65 patients [34 CD, 7 Ulcerative Colitis (UC), 24 others] previously published by O'Brien et al. (2014). Samples were taken from involved (*n* = 59) and uninvolved (*n* = 53) areas on ileal resected specimens and mesenteric lymph nodes (*n* = 53). Cohort 2 is a French cohort of 62 patients (17 CD, 14 UC and 31 non-inflammatory controls). Mucosal biopsies were collected at diagnosis, and represent 45 Peyer's patches and 51 non-inflammatory ileal mucosa. Cohort 3 consisted of 211 CD resected bowel specimens from the REMIND study (Fumery et al., 2017), representing 211 inflamed ileal samples. The main characteristics of the three cohorts are summarized in Table 1.

**Table 1:** Characteristics of patients included in the three cohorts.

		**Cohort 1, (O'Brien et al., 2014)**	**Cohort 2, (unpublished)**	**Cohort 3, (Fumery et al., 2017)**
**All participants**		***n* = 65**	***n* = 62**	***n* = 211**
Disease status	CD UC No-IBD controls	52% 12% 35%	27% 24% 48%	100% 0% 0%
Gender	Males	60%	53%	45%
Age (year)	Median (interquartile range)	45.1 (23–95)^a^	23.6 (9.5–62)^b^	33.8 (25–70)^b^
Smoking habits	Never smokers Past smokers Active smokers	NA	58% 21% 21%	38% 28% 34%
Genotyping	*NOD2* wt *NOD2* mutated^*^	68% 32%	89% 11%	56% 44%
	*ATG16L1* (AA) *ATG16L1* (AG + GG)	18% 82%	29% 70%	21% 79%
	*IRGM* (CC) *IRGM* (CT + TT)	85% 15%	74% 26%	62% 38%
**CD patients only**		***n*** = **34**	***n*** = **17**	***n*** = **211**
Age at diagnosis	A1 (< 17 years) A2 (17–40 years) A3 (>40 years)	16% 59% 25%	55% 45% 0%	10% 80% 10%
Disease Location	L1 (ileum) L2 (colon) L3 (ileocolon) L4 (upper gastrointestinal)	59% 13% 25% 3%	20% 50% 30% 0%	60% 1% 38% 2%
Disease Behavior	B1 (inflamatory) B2 (stricturing) B3 (penetrating)	6% 72% 22%	80% 0% 20%	16% 48% 36%

CD, Crohn's Disease; UC, Ulcerative Colitis; IBD, Inflammatory Bowel Disease;

a at diagnosis;

b at enrolment;

* *one or more of the R702W, G908R, or L1007fs-insC mutations*.

*Cohort 1 and 3: post-surgical resected tissues, cohort 2: endoscopic mucosal biopsies*.

*Montreal Classification is reported for CD patients (Satsangi et al., 2006)*.

All participants provided a written informed consent and the study was approved by the relevant ethic committees in France and Australia [Australian National University Human Ethics Committee approval (ref 2012/596), ACT Health Human Research Ethics Committee approval (ref ETH.5.07.464), AFFSAPS approval (ref IDRCB: 2009-A00205-52) and French ethic committee Hôpital Saint Louis (ref 2009/17)].

### DNA extraction and amplification

For cohort 1, DNA was extracted using DNeasy Blood and Tissue kit (Qiagen) as previously described (O'Brien et al., 2014). For cohort 2, DNAs were extracted using QIAamp DNA mini kit (QIAgen) according to standard protocols. For the third cohort, DNA extraction was performed from specimens preserved in RNAlater, using Trizol reagent (Invitrogen life technologie) with NucleoSpin Tissue (Macherey-Nagel).

DNA quantity and quality were checked for all samples using a Nanodrop ND-1000 spectrophotometer (NanoDrop Technologies, USA). Of the total of 509 DNA samples, 39 with DNA concentrations less than 20 ng/μl were removed. DNA samples were pre-amplified using the GenomiPhi V2 DNA Amplification kit (GE Healthcare) according to the manufacturer's instructions. Briefly, 25 ng of each DNA sample was heat-denatured at 95°C. After addition of the enzyme solution, the sample was incubated at 30°C for 90 min. DNA polymerases were then heat-inactivated at 65°C for 10 min and samples were stored at −20°C until use.

### *Yersinia* identification

**Figure 1:**
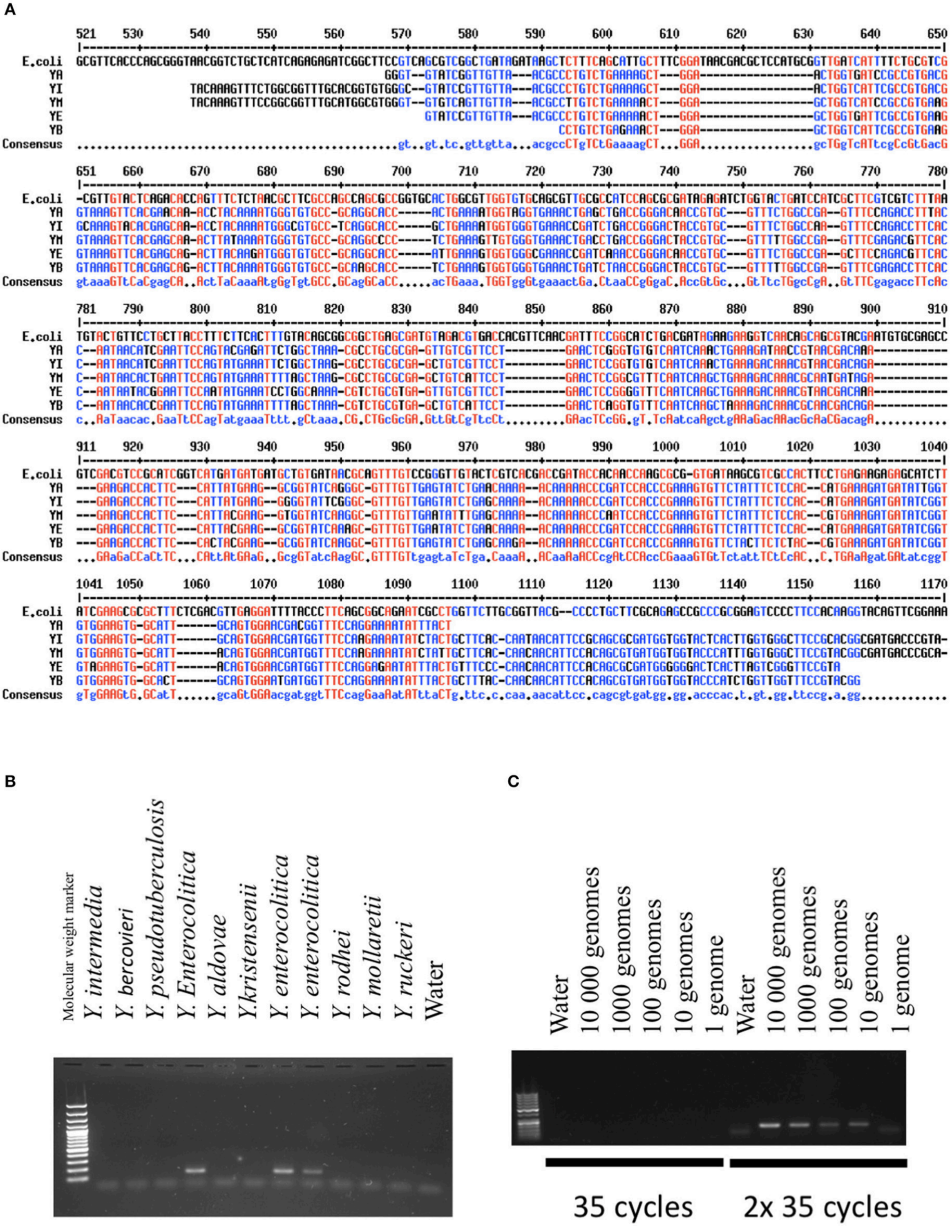
Validation of the PCR methods. **(A)** Comparison of GyrB sequences between different *Yersinia* species and *E.coli*. In red are indicated the nucleic acids present in all species. In blue is indicated the consensus sequence (Corpet, 1998). *YA, Yersinia aldovae; YB, Yersinia bercovieri; YE, Yersinia enterocolitica; YI, Yersinia Intermedia; YM, Yersinia Mollaretii*. **(B)** Specificity of the PCR methods developed for *Y. frederiksenii* (GyrB). DNAs from several species of *Yersinia* were amplified with the PCR technique and loaded on an agarose gel. A band indicates the presence of the specific PCR product specific to *Y. frederiksenii*. **(C)** Sensitivity of the PCR method developed for *Y. enterocolitica* (GyrB). The quantity of amplified DNA is expressed as genome equivalents. The method was highly sensitive when two cycles of 35 amplifications were performed.

Unlike of previous studies, we wanted to identify several *Yersinia* species frequently encountered in contaminated food. We focused on *Yersinia*-specific sequences present in the genome regardless their pathogenicity and ability to differentiate *Yersinia* species. *In silico* analyses directed us to focus on *gyrB*, a gene coding for a subunit of DNA gyrase. Proteins encoded by *gyrB* exhibit considerable variation between species (Mun Huang, 1996). Their substitution rates have been estimated to be 4-fold higher than RNA 16S and *gyrB* has therefore been used to differentiate species and genera phylogenetically. Figure 1A shows the main differences between DNA sequences of different species of *Yersinia* and *E. coli*. In addition to *gyrB*, we determined the presence of *inv*, a virulence gene present in 100% of *Y. pseudotuberculosis* strains (Thoerner et al., 2003), and *ail*, a gene which is a marker of pathogenicity of *Y. enterocolitica*.

PCR assays were performed with 1.2 μl of pre-amplified DNA in a 20 μl volume containing Platinum blue reagent (Invirogen life technologie) together with reverse and forward primers (0.2 μM of each). Our PCR assays were specific for the following six species: *Y. aldovae, Y. bercovieri, Y. enterocolitica, Y. intermedia, Y. mollaretii*, and *Y. pseudotuberculosis*. The primer sequences are provided in Table 2. PCR reactions were carried out using an Eppendorf Mastercycler (Eppendorf, Germany). An initial denaturation was performed at 95°C for 10 min, followed by 35 cycles of 95°C for 30 s, annealing temperature for 30 s and 72°C for 30 s. A second PCR reaction of 35 cycles was performed using 1.2 μl of the first-round PCR products, using the same conditions described above. All PCR reactions included negative controls.

**Table 2:** Primers used for *Yersinia* species identification.

***Yersinia* species**	**Gene name**	**Forward and reverse primers sequences (5′-3′)**	**Size of PCR product (bp)**	**Annealing temp (°C)**
*Y. enterocolitica*	*ail*	ACTCGATGATAACTGGGGAG CCCCCAGTAATCCATAAAGG	170	56
*Y. enterocolitica*	*gyrB*	AACCGATCAAACCGGGACAA AGCTTGATTGAAACCCCGGA	138	60
*Y. pseudotuberculosis*	*inv*	CGGTACGGCTCAAGTTAATCTG CCGTTCTCCAATGTACGTATCC	183	60
*Y. bercovieri*	*gyrB*	CGCAAGCACCTCTGAAAGTG CCGTACGGAAACCAACCAGA	414	60
*Y. mollaretii*	*gyrB*	AACTGACCTGACCGGGACTA GGAAGCCCACCAAATGGGTA	380	60
*Y. intermedia*	*gyrB*	AAGTTTCTGGCGGTTTGCAC CAGATCGGTTTCACCCACCA	138	60
*Y. aldovae*	*gyrB*	GATCCGCCGTGACGGTAAAG GCCCTGATACCGCCTTCATA for nested-PCR:		60
		CGCAGGCACCACTGAAAATG TGACACACCCGAGTTCAGGA	158	60

PCR products were then analyzed on 1.5% agarose gels and stained with SYBR-safe (Invitrogen). Sequencing was subsequently performed on all gel-purified PCR products (QIAquick extraction kit, Qiagen). Bi-allelic direct DNA sequencing was applied using BigDye Terminator v1.1 reagent (Applied Biosystems, USA), on an ABI 3730 automated genetic analyzer and analyzed using the 4peaks software. Sequences were then compared with those present in the NCBI public databases using BLAST. The presence of *Yersinia* was assumed if BLAST returned a homology score with *Yersinia* of at least 95%.

### *NOD2, ATG16L1* and *IRGM* genotyping

Allelic discrimination assays were used to genotype patients and controls for five susceptibility variants: *NOD2* SNPs rs2066844 (assay ID: C-11717468_20), rs2066845 (assay ID: C_11717466_20), and rs2066847 (custom assay); *ATG16L1* SNP rs2241880 (assay ID: C_9095577_20); and *IRGM* SNP rs10065172 (assay ID: C_30593568_10). These SNPs were analyzed using real-time PCR as recommended by the manufacturer (Applied Biosystems, USA). Reactions were carried out using a LightCycler 480 Systems (Roche). An initial denaturation step was performed at 95°C for 10 min, followed by 40 cycles of 92°C for 15 s and 1 min at 60°C.

### Statistics

Comparisons between groups were done using Fisher exact test for quantitative data, combined with a student *T*-test for qualitative data. Tests were performed using Xlstat V1.2. A *P*-value <0.05 was retained as significant.

## Results

### Validation of the PCR methods

Validation of the PCR techniques was based on the analysis of DNA from 10 Yersinia species (*Y. intermedia, Y. bercovieri, Y. pseudotuberculosis, Y. entomophaga, Y. aldovae, Y. frederiksenii, Y. mollaretii, Y. kristensenii, Y. ruckeri, and Y. rohdei*) kindly provided by Elisabeth Carniel from the international reference center of Yersinia, Institut Pasteur, Paris, France. PCRs were performed using 5 ng of DNA (corresponding to ~10^6^ genomes). All species except Y. rohdei were detected by the selected primers. Figure 1B shows the results obtained with gyrB specific primers for Y. *enterocolitica*.

To evaluate the sensitivity of the PCR methods, we serially diluted the DNA samples between 1 to 10,000 genomes based on an estimated genome weight of ~4.7 × 10^−6^ ng. The serial DNA dilutions underwent 35 PCR cycles, but no PCR products were observed in the majority of cases (Figure 1C). We thus performed a second round of 35 cycles, which allowed us to detect the bacteria. In these conditions, the thresholds were estimated between 1 and 100 equivalent genomes.

We also determined if the PCR techniques were efficient in detecting *Yersinia* in a DNA sample containing human genomic DNA. The presence of eukaryotic DNA did not alter the sensitivity of the methods (data not shown). We also confirmed that genomic pre-amplification did not significantly change the capacity of the PCR to detect *Yersinia* DNA (data not shown). Of note, when performed on human samples, the specificities of the PCR methods were lower than previously described with purified *Yersinia* DNA only. Indeed, several non-specific PCR products were evident for ileal samples, indicating that sequencing was always necessary to validate the presence of *Yersinia*-specific sequences in tissues.

Ultimately, we retained the PCR assays for *Y. aldovae, Y. bercovieri, Y. enterocolitica, Y. intermedia, Y. mollaretii*, and *Y. pseudotuberculosis*. For the other species, the sensitivity and/or specificity of the techniques were deemed to be insufficient.

### *Yersinia* in CD and controls

We analyzed a total of 140 samples from 76 controls (ischemic colitis, intestinal obstructions, diverticular diseases and others) (Table 3). Seventy nine samples were from cohort 1 and consisted of mucosal tissues and lymph nodes. We found 6/54 specimens and 3/25 lymph nodes positive for *Yersinia* corresponding to a total of 11%. When several samples were available for the same individual, some were found positive, while others were not. This result indicated that the status of a given individual was highly dependent on the number of samples studied but also on the number of PCR reactions conducted on the same DNA sample (data not shown). In cohort 2, we found *Yersinia* DNA in 2/26 Peyer's patches (8%) and 0/35 mucosal biopsies, corresponding to a total of 3%. Thus a total of 8% of positive samples were found in the combined cohorts.

**Table 3:** Detection of *Yersinia* species in samples from the three cohorts.

	**Cohort 1 (O'Brien et al.**, **2014****)**						**Cohort 2 (unpublished)**				**Cohort 3 (Fumery et al., 2017)**
**Disease status**	**CD (*****n*** = **34)**			**controls (*****n*** = **31)**			**CD (*****n*** = **17)**		**controls (*****n*** = **45)**		**CD (*n* = 211)**
**Sample's area *(n)***	**NIA (28)**	**IA (30)**	**LN (28)**	**NIA (25)**	**IA (29)**	**LN (25)**	**PP (17)**	**IM (16)**	**PP (26)**	**IM (35)**	**IA (211)**
*gyrB* for *YE*	0	3	1	3	2	2	0	0	2	0	9
*ail* for *YE*	0	1	0	0	0	0	0	0	0	0	2
*inv* for *YP*	1	0	0	0	0	1	1	0	0	0	2
*gyrB* for *YB*	0	0	0	0	0	0	0	0	0	0	0
*gyrB* for *YM*	0	0	0	1	0	0	0	0	0	0	1
*gyrB* for *YA*	0	0	0	0	0	0	0	0	0	0	1
*gyrB* for *YI*	1	0	0	2	0	0	0	0	0	0	2
Positive samples	2 (7%)	4 (13%)	1 (4%)	4^*^ (16%)	2 (7%)	3 (12%)	1 (6%)	0 (0%)	2 (7%)	0 (0%)	17 (8%)

*CD, Crohn's Disease; NIA, Non-inflammatory area; IA, inflammatory area; LN, lymph node; PP, Peyer's patch; IM, ileal mucosa; YE, Y. enterocolitica; YP, Y. pseudotuberculosis; YB, Y.bercovieri; YM, Y. molaretii; YA, Y. aldovae; YI, Y. intermedia*.

* *one patient was positive for 3 Yersinia species*.

In cohorts 1 and 2, *Yersinia* species identified in controls were *Y. enterocolitica* (*n* = 9), *Y. intermedia* (*n* = 2), *Y. pseudotuberculosis* (*n* = 1), and *Y. molaretii* (*n* = 1). No differences were found between inflammatory and non-inflammatory controls.

In CD patients (all cohorts), as for the control group, biopsies were less often positive (1/33, 3%) than resected specimens or lymph nodes (24/297, 8%). On average, no clear difference was observed between CD cases and inflammatory or non-inflammatory controls. The *Yersinia* species encountered in CD patients were *Y. enterocolitica* (*n* = 13), *Y. intermedia* (*n* = 3), *Y. pseudotuberculosis* (*n* = 4), *Y. molaretii* (*n* = 1), and *Y. aldovae* (*n* = 1) (Table 3). These results are similar to those of the control groups. Of note, only 3/13 *Y. enterocolitica* contained the *ail* gene suggesting that most of the strains present in the mucosa were not fully virulent (Sihvonen et al., 2011).

### Impact of genetic and clinical parameters on the presence of *Yersinia* species

To look for an association between clinical, genetic or therapeutic parameters and the presence of *Yersinia* in CD patients we analyzed the data from cohorts 1 and 3 which were fully phenotyped and genotyped (Table 4). Positivity rates were roughly identical when gender, Montreal classification [age at diagnosis, behavior, and location) (Satsangi et al., 2006)] and genotypes were included in the analysis. Most of the therapeutic regimens also did not affect the frequency of detection, however a borderline association between the use of immunosupressants and the absence of *Yersinia* was found in cohorts 1 (*P* = 0.08) and 3 (*P* = 0,08). This association reached significance when pooling the two cohorts (*P* = 0.035).

**Table 4:** Presence of *Yersinia* in ileal samples according to clinical and genetic classification of CD patients.

		**Cohort 1**			**Cohort 3**		
		**Total**	***Yersinia* +n (%)**	***P-value***	**Total**	***Yersinia* +n (%)**	***P-value***
Gender	Female Male	13 20	3 (23) 4 (20)	*1*	109 90	10 (9) 7 (8)	*0.80*
Age at diagnosis	A1 (< 17 years)	5	2 (40)		20	1 (5)	
	A2 (17–40 years)	19	4 (21)		159	13 (8)	
	A3 (>40 years)	8	1 (12)	*0.60*	20	3 (15)	*0.63*
Disease behavior	B1 (inflammatory)	2	0 (0)		32	4 (13)	
	B2 (stricturing)	23	5 (22)		96	5 (5)	
	B3 (penetrating)	7	2 (29)	*1*	71	8 (11)	*0.23*
Disease location	L1 (ileum)	19	4 (21)		120	8 (7)	
	L2 (colon)	4	1 (25)		1	0 (0)	
	L3 (ileocolon)	8	2 (25)		75	9 (12)	
	L4 (upper gastrointestinal)	1	0 (0)	*1*	3	0 (0)	*0.45*
Genotype	*NOD2* wt *NOD2* mutated^*^	20 14	4 (20) 3 (21)	*0.92*	118 93	11 (9) 6 (6)	*0.61*
	*ATG16L1* (AA) *ATG16L1* (AG + GG)	6 28	1 (17) 6 (21)	*1*	45 165	6 (13) 11 (7)	*0.21*
	*IRGM* (CC) *IRGM* (CT + TT)	28 6	6 (21) 1 (17)	*1*	130 80	10 (8) 7 (9)	*0.80*
Medications at surgery	Antibiotics No antibiotics	9 22	1 (11) 5 (22)	*0.64*	65 134	7 (11) 10 (7)	*0.43*
	Anti-TNF No anti-TNF	8 22	2 (25) 4 (18)	*0.64*	99 99	6 (6) 11 (11)	*0.31*
	Immunosuppressants No immunosuppressants	15 14	1 (7) 5 (36)	*0.08*	60 138	2 (3) 15 (11)	*0.08*
	Corticosteroids No corticosteroids	17 13	1 (6) 5 (38)	*0.06*	67 131	9 (13) 8 (6)	*0.10*
	5-ASA No 5-ASA	7 22	2 (29) 4 (18)	*0.61*	16 183	2 (12) 15 (8)	*0.63*
Rutgeerts score	i0 + i1 i2 + i3 + i4	NA			87 70	10 (11) 4 (6)	*0.26*

* *one or more of the R702W, G908R or L1007fs-insC mutations; 5-ASA, 5 aminosalycilates*.

Most patients from cohort 3 were followed prospectively for at least 6 months after surgery. It was thus possible to evaluate the prognostic value of the presence of *Yersinia* at surgery on disease recurrence risk. The Rutgeerts endoscopic severity index, based on the presence of small bowel ulcerations, is widely used to monitor the presence of post-operative recurrence (*i* > 1) or not (*i* < 2). The Rutgeerts index calculated at least 6 months after surgery was not associated with the presence of *Yersinia* at surgery, suggesting that a sample positive for *Yersinia* is not predictive of an early recurrence.

## Discussion

This study is the largest report of the presence of *Yersinia* in the human ileum. It is based on a total of 470 samples, taken from controls (140 samples) and CD patients (330 samples). The samples consisted of non-inflammatory ileal mucosa, Peyer's patches, resected bowel specimens, and lymph nodes. We looked for six different species of *Yersinia* with a highly sensitive PCR method, and sequenced all of the PCR products to confirm the presence of *Yersinia-*specific DNA.

This study proposes a new set of PCR tests based on the amplification of *gyrB* sequences, capable of identifying *Y. aldovae, Y. bercovieri Y. enterocolitica, Y. intermedia*, and *Y. mollaretii*. Despite a very high specificity of the tests *in vitro*, PCR products must always be sequenced to confirm the presence of *Yersinia* in tissues, because non-specific sequences are frequently encountered *in vivo*. These tests are highly sensitive, with detection thresholds between 1 and 100 bacteria per sample.

Given the low thresholds of bacterial detection, sampling plays an important role in the assay's results: increasing the number of samples tested increases the number of positive results on a per patient basis. This point is well illustrated by cohort 1 where three samples were tested for most patients with a positivity of 21% at the patient level but only 10% at the sample level. As a consequence, it is possible that *Yersinia* species could be found in the ileum of many people (may be all) if one performs enough tests. As a whole, the percentage and significance of negative results in patients remains to be evaluated. The concordance between this new test and other methods like serological tests or immunohistochemistry methods also needs to be explored.

If our work suggests that *Yersinia* carriage is common, it also indicates that there are generally low numbers in the ileum. This result is consistent with the fact that *Yersinia* are rarely found in stool and mucosal samples from either CD patients or controls in gut microbiota analyses (Sihvonen et al., 2011). These are designed to explore the dominant species, and are not appropriate for detecting low abundance of *Yersinia* species.

Low bacterial loads are also in line with a common exposure to the few bacteria assumed to be present in contaminated food. The main bacteria encountered in the ileum are *Y. enterocolitica* which represent about 2/3 of the identified bacteria. *Y. pseudotuberculosis* and *Y. intermedia* are the second most commonly found here. Even if we did not test all the panel of *Yersinia* species, these relative frequencies seem to reflect the relative frequencies of *Yersinia* species in food products (Özdemir and Arslan, 2015). Among *Y. enterocolitica*, most of the strains did not carry the *ail* gene indicating that they are not fully virulent. In other words, bacteria appear to be mainly non-virulent, or transient (allochthonous), and their retrieval from the gut may simply be a marker of food contamination. Additional works looking at a relationship between food habits and the presence of Yersinia in the gut would help to resolve this question.

On the other hand, looking at *Yersinia* species as inoffensive bystanders passively transported by the luminal flow can be questioned. Yersinia species are able to survive in the ileum due to their facultative anaerobic properties and tolerance to the bile acids (Bottone, 1997). They are able to interact with the gut associated lymphoid tissue and we confirm here the tropism of *Yersinia* for M-cells, the bacteria being mainly found in superficial biopsies centered on Peyer's patches. However, superficial biopsies tended to be positive less often than resected bowel specimens (*P* = 0.058). This may be related to technical differences in sample collection and storage. But it may also indicate that the bacteria are more commonly detectable within the inner intestinal layers. This point is in accordance with a previous study showing the presence of *Yersinia* in the submucosal layer of the intestine (Chiodini et al., 2013). The relatively high frequency of bacteria in mesenteric lymph nodes also supports this idea. If true, this finding indicates that Yersinia, even low virulence strains, are able to translocate through the gut epithelial barrier and putatively the gut-vascular barrier (Spadoni et al., 2015).

It is important to note that bacteria were equally frequent in non-inflammatory, and inflammatory controls and CD patients, and that *Yersinia* could be found in inflammatory and non-inflammatory parts of the ileum in CD patients. Thus, CD cannot be seen as an infection by *Yersinia* species: the disease is not defined by its presence in the intestinal tissues. However, it cannot be dismounted that CD patients have an abnormal response toward *Yersinia*. Indeed, *Yersinia* have been associated with long-term sequelae after acute infection in mice, including destruction of the lymphatic network and loss of antigen tolerance, similar to CD (Fonseca et al., 2015). Furthermore, CD is characterized by increased adipose tissue around the lesions (fat wrapping). White adipose tissue has been recently shown to play a key role in the long-term memory of infection by *Yersinia* (Han et al., 2017). Thus CD could be characterized by an excessive response toward *Yersinia*. The abnormal inflammatory response to *Yersinia* in mice carrying either *Nod2* or *Atg16l1* CD-associated mutations (Meinzer et al., 2008; Murthy et al., 2014) further supports this idea.

This work failed to detect an association between phenotypic or genotypic subgroups of patients. Mutations in *NOD2* (a gene involved in the innate immunity and bacterial sensing) or *ATG16L1* and *IRGM*, (two genes involved in autophagy and xenophagy) are not associated with *Yersinia* positivity despite their roles in the host response to the bacteria. The age at onset is also not associated with the presence of *Yersinia*. It is more difficult to demonstrate an association between disease location and behavior (L and B values of the Montreal classification). Indeed, fistulizing, and penetrating ileal diseases are over-represented in surgical patients, which represented the largest contingent of the studied cohorts.

Common medications used to treat CD patients, including antibiotics, are not associated with the presence of *Yersinia*. This finding is not surprising if we consider that they are often administered prior to surgery. As a result, bacteria, including dead cells, remain detectable by PCR. Steroids, immunosuppressants or anti-TNF antibodies could be expected to decrease bacterial clearance and promote *Yersinia* detection. However, the presence of bacteria at very low rates in the mucosa must be distinguished from an invasive infection and the impact of immunosuppressive agents is likely to be different in these two situations. At the opposite, immunosuppressants tended to be associated with a lower rate of positive samples in two cohorts. An hypothesis to explain this finding is that azathioprine (which is the most commonly used immunosuppressant in CD), modulates intracellular GTP levels and thus interferes with the function of Rac1, a Rho GTPase (Tiede et al., 2003). Rho GTPase activities are important for bacterial internalization by host cells and subsequent dissemination and survival of bacteria (Schweer et al., 2013). A defect of bacterial internalization could be disadvantageous for bacterial survival and azathioprine could thus have a beneficial role in CD, not only via its immunosuppressive properties, but also via the reduction of *Yersinia* burden in the gut mucosa.

In summary, this work provides for the first time the demonstration that *Yersinia* species are common in human ileum. The presence of different species seems to be proportional to assumed rates of exposure in contaminated foods. *Yersinia* are found in the same proportions in controls and CD patients, whatever their clinical presentation. It is important now to characterize the mucosal immune response toward *Yersinia* species in CD patients.

## Author contributions

GL, AD, and J-PH were involved in study design, data acquisition, analyses and writing. CO, PP were involved in study design, data acquisition, and analyses for cohort 1. JV, J-PH, DC-H, and MA were involved in data acquisition for cohort 2. PS, XT, SN, MB, CA, and The REMIND Group were involved in study design, data acquisition and analyses for cohort 3. NB, MR, and AD were involved in participants genotyping PCR analyses.

### Conflict of interest statement

The authors declare that the research was conducted in the absence of any commercial or financial relationships that could be construed as a potential conflict of interest.
